# Psychosocial factors and dietary patterns in metabolic and bariatric surgery: analyzing anxiety, depression, and hedonic hunger

**DOI:** 10.1007/s40519-025-01778-5

**Published:** 2025-08-16

**Authors:** Beatriz Vieira, Zélia Santos, Rui Ribeiro, Octávio Viveiros, Carina Rossoni, Elisabete Carolino, Filipa Novais

**Affiliations:** 1 Hospital Lusíadas Amadora, Amadora, Portugal; 2 Faculdade de Medicina, Universidade de Lisboa, Lisboa, Portugal; 3 H&TRC-Health&Technology Research Center, ESTeSL-Escola Superior de Tecnologia da Saúde, Instituto Politécnico de Lisboa, Lisboa, Portugal; 4 Coordenador Do Centro Multidisciplinar de Tratamento de Obesidade, Hospital Lusíadas Amadora, Amadora, Portugal; 5 Serviço de Neurociências E Saúde Mental, Hospital Santa Maria-Centro Hospitalar Universitário Lisboa Norte, Lisboa, Portugal

**Keywords:** Metabolic and bariatric surgery, Hedonic hunger, Anxiety, Depression, Mediterranean Diet

## Abstract

**Background:**

Scientific evidence has demonstrated that psychosocial factors can influence hedonic hunger (HH). Moreover, HH can be a predictor of metabolic and bariatric surgery (MBS) outcomes. The Mediterranean Diet (MD) has been used as a model approach for managing excess weight. In this study, we assessed the association between anxiety and depression levels, HH, and adherence to MD in candidates to MBS and post-MBS subjects.

**Methods:**

This was a cross-sectional observational study that included individuals who were candidates and who were submitted to MBS. Data analysis included anthropometric measurements, assessment of anxiety and depression levels, HH, and adherence to MD.

**Results:**

Of the 64 included individuals, 68.8% were female, with a mean age of 48.39 years. A significant positive correlation was observed between HH and anxiety and depression levels. No statistically significant relationship was found between adherence to MD and HH, nor with anxiety and depression levels.

**Conclusion:**

Psychological status largely contributes to weight control. Therefore, patients should be carefully assessed before and after surgery to ensure treatment and adequate follow-up. Future studies are needed to better determine the complex relationship between psychological factors, HH, food intake, and weight.

*Level V:* Cross-sectional observational study.

## Introduction

Metabolic and bariatric surgery (MBS) has been described as an effective method for treating obesity and is indicated for individuals with a body mass index (BMI) of 35 kg/m^2^ or above, regardless of the presence, absence, or severity of comorbidities [1–4].

Scientific evidence has demonstrated that psychosocial factors impact the development of overweight. Difficulty in managing and regulating emotions and chronic exposure to episodes of stress seems to contribute to weight gain and hinder weight loss. Furthermore, eating is associated with a feeling of pleasure, which often functions as a maladaptive coping mechanism that may result in a loss of control over the amount of food consumed [5].

A high intake of foods with a high energy density and high sugar and fat content—considered foods with high palatability—is identified as one of the main factors contributing to the global increase in obesity [7, 8].

Hedonic hunger (HH) refers to the appetite for foods with high palatability, which is motivated by the constant availability of these foods in the environment [6]. This behavior is influenced by neurobiological and psychological mechanisms, including reward sensitivity and emotional associations with food intake. The inclusion of hedonic hunger in this study is essential to better understand the complex eating behaviors that challenge weight management, particularly in individuals undergoing MBS, where such drives may impact both food intake and surgical outcomes [7].

The Mediterranean Diet (MD) has been used as a model approach for the prevention and management of excess weight [9, 10]. Gastaldo et al. found that adherence to MD may be directly related to weight loss, both before and after MBS [11].

In recent years, the role of diet, particularly MD, has been a focus of research and debate in the development of mental disorders [12, 13]. According to Parletta et al., participants who adhered to MD reported significant improvement in depressive symptoms [14].

However, there is still limited evidence exploring the interplay between hedonic hunger, mental health (specifically anxiety and depression), and adherence to the Mediterranean Diet in the context of MBS. Investigating these factors in an integrated manner may provide a broader understanding of psychosocial determinants of eating behavior before and after surgery.

This study aimed to determine the most important factors associated with HH, focusing on anxiety and depression status and adherence to MD, in candidates and post-MBS subjects.

## Materials and methods

### Sample

The study included a convenience sample with 32 candidates for MBS and 32 post-MBS individuals with one-anastomosis gastric bypass (OAGB) or single sleeve ileal anastomosis (SASI). Participants were enrolled if they had undergone surgery at least 12 months before and up to 24 months. The inclusion criteria comprised individuals aged 18 years or older and younger than 65 years. Individuals who had undergone revision surgery, patients with cancer, individuals with eating behavior disorders, pregnant women, individuals with major pathology, those with severe cases of anxiety and depression, and those on psychotropic medication were excluded.

### Procedures

**Fig. 1:**
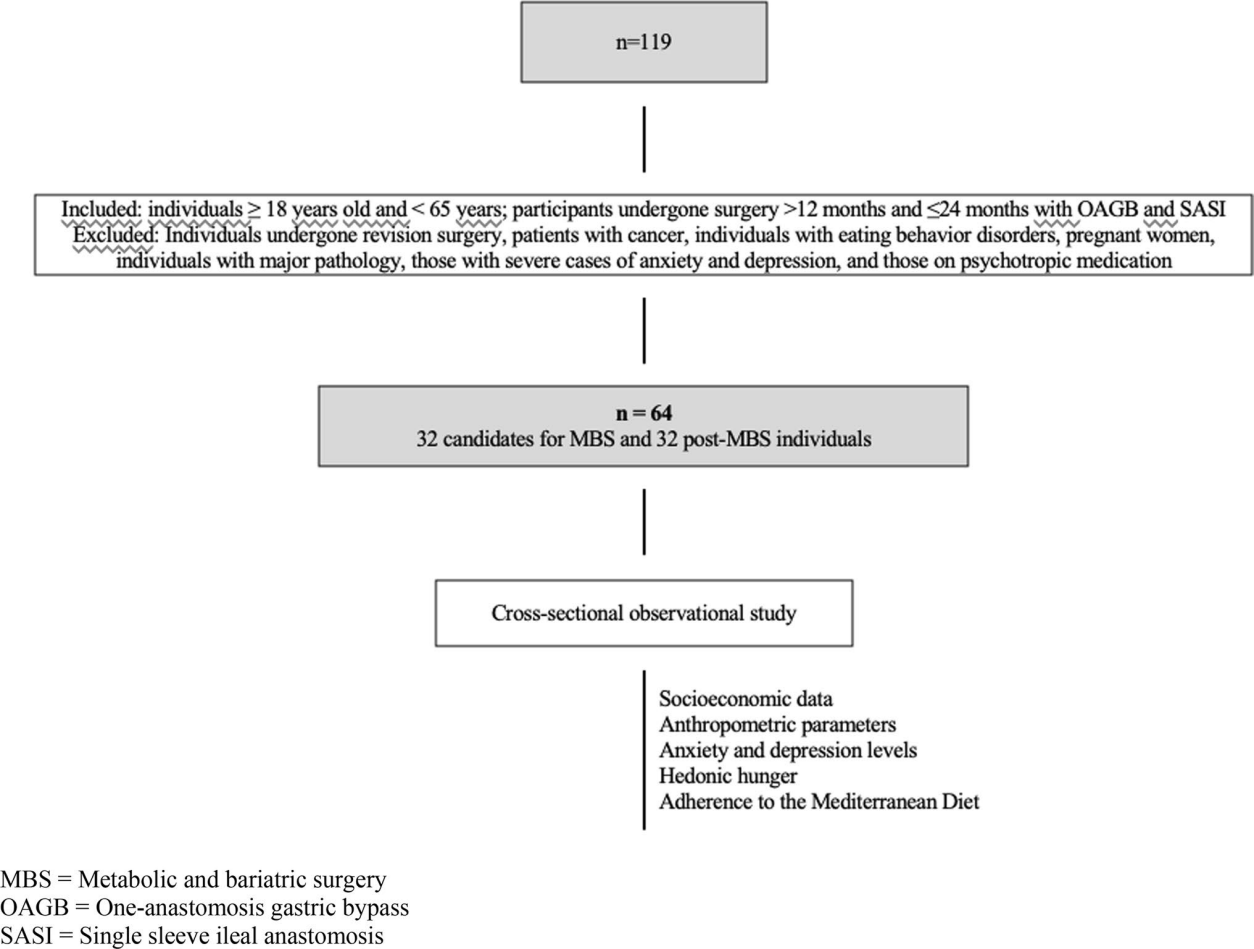
Flowchart of the procedures. MBS: metabolic and bariatric surgery. OAGB: one-anastomosis gastric bypass. SASI: single sleeve ileal anastomosis

This was a cross-sectional observational study (Fig. 1). To collect data, an online questionnaire was sent to participants, with the aim of analyzing socioeconomic data and anthropometric parameters. The questionnaire also included instruments to assess anxiety and depression levels, HH, and MD adherence. All individuals underwent a clinical assessment, conducted specifically by the psychology team, to screen for and exclude those with eating behavior disorders and severe cases of anxiety and depression. The demographic characteristics of both groups, including age and sex, were compared.

#### Anxiety and depression levels

The Hospital Anxiety and Depression Scale (HADS) tool validated for the Portuguese population was used to evaluate anxiety and depression levels. Values of 7 or less are not considered a sign of disturbance, values between 8 and 10 are interpreted as cases in which disturbance may exist, and values of 11 or above are considered cases with the presence of a clinically significant disturbance [15, 16].

#### Hedonic hunger

The Power of Food Scale (PFS) questionnaire, developed by Lowe et al. and validated for the Portuguese population by Ribeiro et al., was used to assess HH. The total score is calculated by averaging the item scores, with higher values indicating a stronger appetite for foods with high palatability [17, 18].

This tool is based on the premise that appetite increases in environments where palatable foods are available. In this way, the PFS measures the impact of foods with high palatability across three levels of proximity: the first presupposes the wide availability of palatable foods in the environment but not physically present (available foods); the second involves reactions to foods that are physically present but never tasted (present foods); and the third encompasses reactions to foods with high palatability when they are tasted for the first time but not consumed (tasted foods) [17, 18].

#### Adherence to the Mediterranean Diet

The Prevención com Dieta Mediterranea (PREDIMED) questionnaire, which was developed in Spain to test the efficacy of MD for the primary prevention of cardiovascular disease and validated for the Portuguese population, was used to assess adherence to MD. A final score of 10 or above indicates good adherence to the Mediterranean dietary pattern [19].

We used the photographic models from the Photographic Food Quantification Manual of the National Food and Physical Activity Survey to answer the PREDIMED questions [20].

### Statistical analysis

Data analysis and processing were performed using IBM SPSS^®^ 27.0 for Windows.

The results were considered significant at a 5% significance level.

To characterize the sample, frequency analysis (*n*, %) was used for qualitative data, and the minimum, maximum, mean, and standard deviation were used for quantitative data.

The Shapiro–Wilk test was used to assess data normality.

To compare quantitative variables between two independent groups, the *t*-test for two independent samples or the Mann–Whitney *U* test was used, depending on the assumption of normality.

To study the relationship between two quantitative variables, Pearson or Spearman correlation coefficients were used, depending on the verification of the assumption of normality.

To explore the relationship between two qualitative variables, the Chi-square test was used when the applicability assumptions were met or, when not, the Chi-square test was performed by Monte Carlo simulation or Fisher’s exact test.

## Results

The individuals included in the study had a mean age of 48.39 ± 9.56 years. The sample predominantly consisted of female participants (68.8%). We tested and found that age did not differ significantly between groups (*p = *0.6060). However, a significant difference was observed regarding sex (*p = *0.031), with a higher proportion of males in the group of post-MBS individuals. The mean BMI was 42.6 ± 5.6 kg/m^2^ in the MBS candidates and 25.1 ± 3.2 kg/m^2^ in the group of post-MBS individuals.

### Anxiety and depression levels

Regarding anxiety levels, 50% of the total sample did not present any disturbance (Table 1).

**Table 1:** Characterization of anxiety levels

		MBS candidates	Post-MBS subjects	Total	Chi-square	Number of degrees of freedom	*p*
		*n* (%)	*n* (%)	*n *(%)			
HADS-anxiety	No disturbance	16 (50.0%)	16 (50.0%)	32 (50.0%)	1.247	2	0.536
	Possible disturbance	4 (12.5%)	7 (21.9%)	11 (17.2%)			
	Clinically significant disturbance	12 (37.5%)	9 (28.1%)	21 (32.8%)			

HADS: Hospital Anxiety and Depression Scale; MBS: metabolic and bariatric surgery

Regarding depression levels, 76.6% did not experience any disturbance (Table 2). The distribution of depression differed significantly between groups (*p = *0.032).

**Table 2:** Characterization of depression levels

					Chi-square test by Monte Carlo simulation		
		MBS candidates	Post-MBS subjects	Total	*p*	95% confidence interval for *p*	
		*n* (%)	*n* (%)	*n* (%)		Lower limit	Upper limit
HADS-depression	No disturbance	20 (62.5%)	29 (90.6%)	49 (76.6%)	**0.032**	0.028	0.035
	Possible disturbance	6 (18.8%)	2 (6.3%)	8 (12.5%)			
	Clinically significant disturbance	6 (18.8%)	1 (3.1%)	7 (10.90%)			

HADS: Hospital Anxiety and Depression Scale; MBS: metabolic and bariatric surgery

No association was observed between age and anxiety (*p = *0.373) or depression (*p = *0.054) levels. Similarly, no association was found between sex and anxiety (*p = *0.1390) or depression (*p = *0.4553) levels. According to Table 3, no relationship was observed between anxiety and depression levels and BMI.

**Table 3:** Relationship between anxiety and depression levels and BMI

			IMC
MBS candidates	HADS-anxiety	Pearson correlation coefficient	− 0.016
		*p*	0.930
	HADS-depression	Pearson correlation coefficient	− 0.093
		*p*	0.612
Post-MBS subjects	HADS-anxiety	Pearson correlation coefficient	− 0.020
		*p*	0.912
	HADS-depression	Pearson correlation coefficient	0.179
		*p*	0.327

HADS: Hospital Anxiety and Depression Scale; MBS: metabolic and bariatric surgery

### Hedonic hunger

The distribution of HH levels did not significantly differ between the groups of individuals (Table 4). No significant difference in hedonic hunger levels was observed between males and females (*p = *0.4545), suggesting that sex is not a confounding factor in this relationship. Additionally, no association was found between hedonic hunger levels and age (*p = *0.957).

**Table 4:** Characterization of hedonic hunger levels

	MBS candidates		Post-MBS subjects		*T*-test		
	Minimum–maximum	Mean ± standard deviation	Minimum–maximum	Mean ± standard deviation	*t*	df	*p*
PFS total	1–5	2.83 ± 0.87	1–5	2.60 ± 1.03	0.942	62	0.350
PFS available foods	1–4	2.59 ± 1	1–5	2.45 ± 1.17	0.514	62	0.609
PFS present foods	1–5	3.17 ± 1	1–5	2.81 ± 1.09	1.373	62	0.175
PFS tasted foods	1–5	2.84 ± 0.85	1–5	2.62 ± 1.03	0.927	62	0.358

MBS: metabolic and bariatric surgery; PFS: Power of Food Scale

In this study, no relationship was observed between BMI and HH or between weight loss percentage and HH (Table 5).

**Table 5:** Relationship between hedonic hunger levels and weight loss percentage

		Weight loss percentage
PFS total	Pearson correlation coefficient	− 0.195
	*p*	0.284
PFS available foods	Pearson correlation coefficient	− 0.237
	*p*	0.191
PFS present foods	Pearson correlation coefficient	− 0.135
	*p*	0.461
PFS tasted foods	Pearson correlation coefficient	− 0.146
	*p*	0.424

PFS: Power of Food Scale

### Relationship between anxiety and depression levels and hedonic hunger

When analyzing the sample by groups, no relationship was observed between HH and anxiety and depression levels in the candidates for MBS (Table 6).

**Table 6:** Relationship between anxiety and depression levels and hedonic hunger

			PFS total	PFS available foods	PFS present foods	PFS tasted foods
MBS candidates	HADS-anxiety	Pearson correlation coefficient	0.104	0.108	0.112	0.016
		*p*	0.571	0.557	0.543	0.929
	HADS-depression	Pearson correlation coefficient	0.247	0.253	0.196	0.133
		*p*	0.173	0.163	0.283	0.468
Post-MBS subjects	HADS-anxiety	Pearson correlation coefficient	0.644	0.623	0.649	0.525
		*p*	** < 0.001**	** < 0.001**	** < 0.001**	**0.002**
	HADS-depression	Pearson correlation coefficient	0.435	0.401	0.479	0.425
		*p*	**0.013**	**0.023**	**0.006**	**0.015**

Bold: The results were considered significant at a 5% significance level (*p* < 0.05)

HADS: Hospital Anxiety and Depression Scale; MBS: metabolic and bariatric surgery; PFS: Power of Food Scale

In the group of post-MBS individuals, a moderate positive correlation was found between anxiety levels and HH (*p = * < 0.001), as well as with the subscales of the PFS Available Foods (*p = * < 0.001), Present Foods (*p = * < 0.001) and Tasted Foods (*p = *0.002).

**Fig. 2:**
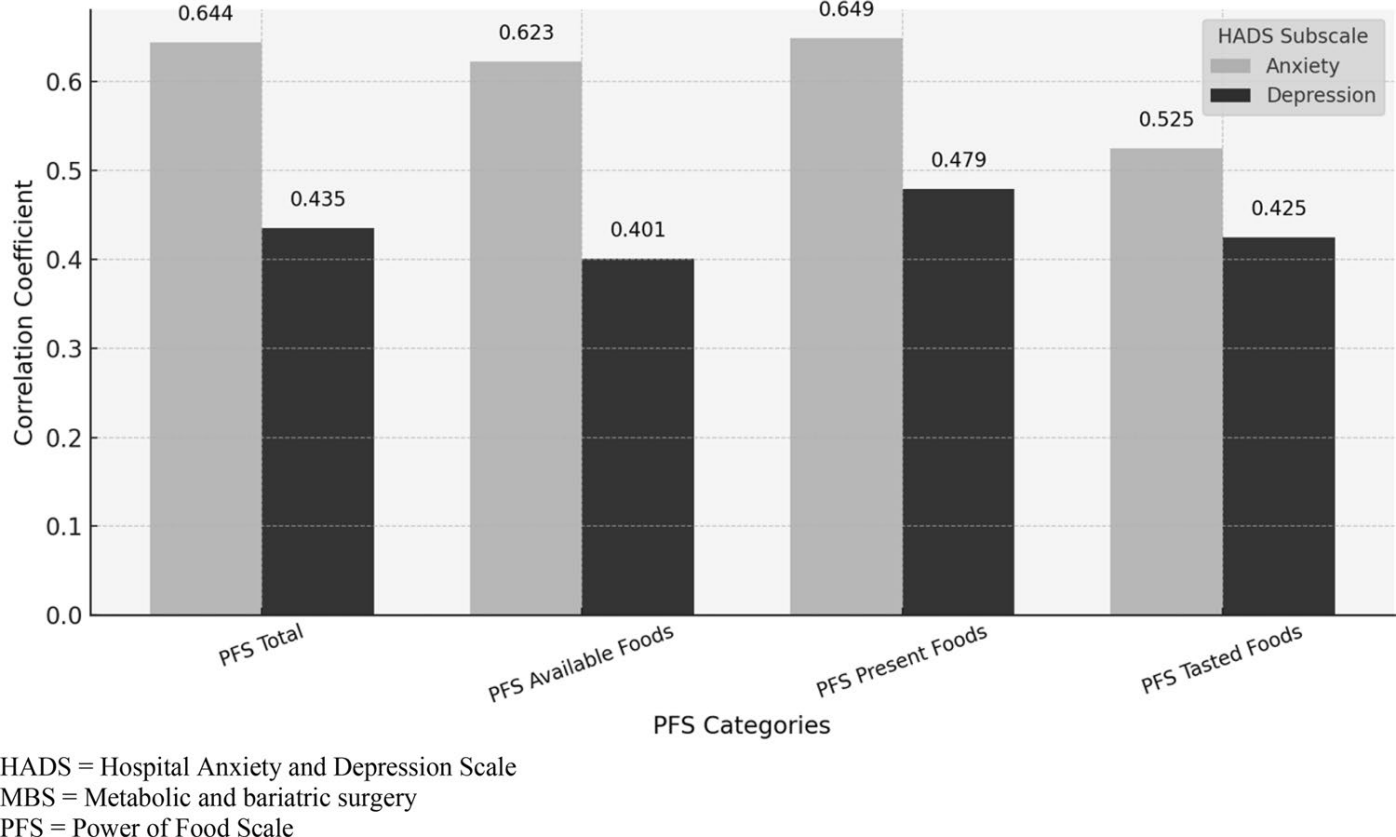
Relationship between anxiety and depression levels and hedonic hunger in the group of post-MBS subjects. HADS Hospital Anxiety and Depression Scale; MBS: metabolic and bariatric surgery; PFS: Power of Food Scale

In addition, in the group of post-MBS individuals (Fig. 2), a positive correlation of weak intensity was observed between depression levels and HH (*p = *0.013), as well as with the PFS subscales Available Foods (*p = *0.023), Present Foods (*p = *0.006) and Tasted Foods (*p = *0.015).

### Adherence to DM

It was found that 76.6% did not have good adherence to MD (Table 7). No association was observed between adherence to MD and age (*p = *0.077). No association was observed between adherence to MD and sex (*p = *0.087).

**Table 7:** Characterization of adherence to DM

		Predimed		Chi-square test		
		Good adherence to MD	Lack of adherence to MD	value	df	*p*
MBS candidates	n (%)	7 (21.9%)	25 (78.1%)	0.087	1	0.768
Post-MBS subjects	n (%)	8 (25.0%)	24 (75.0%)			
Total	n (%)	15 (23.4%)	49 (76.6%)			

MBS: metabolic and bariatric surgery; MD: Mediterranean Diet

No relationship was observed between adherence to MD and BMI or between adherence to MD and weight loss percentage. However, individuals with good adherence to MD showed a higher weight loss percentage (Table 8).

**Table 8:** Relationship between adherence to MD levels and weight loss percentage

	Predimed	*n*	Order average	Mann–Whitney *U* Test	*p*
Weight loss percentage	Good adherence to MD	8	18.25	82	0.542
	Lack of adherence to MD	24	15.92		

MD: Mediterranean Diet

In this study, no relationship was observed between adherence to the Mediterranean dietary pattern and anxiety and depression levels or between adherence to MD and HH.

## Discussion

The distribution of depression significantly differed between the groups, with a higher percentage of clinically significant depression in the MBS candidates. High-quality evidence proves that the score of depressive symptoms can be significantly improved after bariatric surgery within a 2-year follow-up period [21].

The main objective of MBS is to induce significant weight loss and improve obesity-related comorbidities. However, improvements in psychological well-being and the quality of life of individuals with obesity have also been described in the literature [2, 22].

Given the cross-sectional nature of this study, it is not possible to conclude that MBS led to improvements in depression levels. However, several studies have shown that MBS appears to be associated with a reduction in depression and anxiety levels, at least in the first 2–3 years after surgery [23–26].

In this investigation, the distribution of HH levels did not significantly differ between the groups of individuals. Barstard et al. noted a statistically nonsignificant reduction in HH levels after MBS (gastric sleeve and gastric bypass) [27]. Schultes et al. found that post-MBS individuals had lower levels of HH than those with obesity [28]. Makaronidis et al. similarly observed a reduction in HH in post-MBS (gastric bypass) individuals [29]. However, the sample size of this study may be underpowered, which is why this study also encourages the conduct of further research in the future.

No relationship was observed between BMI and HH. Scientific studies on the association between BMI and HH are contradictory. While some studies have presented results consistent with this study [30–32], other authors have found a positive association between BMI and HH [33–36].

No significant association was observed between percentage weight loss and HH. However, a slight trend was observed in the sample, indicating that a higher percentage of weight loss might be related to a decrease in HH levels. These findings align with those of other studies. Aukan et al. found that weight loss appears to be associated with a reduction in HH, regardless of the intervention used (very low energy diet or MBS) [37]. Makaronidis et al. noted that lower HH levels appear to be associated with the percentage of weight lost after MBS [29]. Ribeiro et al. also concluded that HH can be predictive of MBS outcomes and is inversely related to the percentage of weight loss after surgery [38].

The literature has described that stress episodes are associated with increased appetite and that mental condition influences the eating habits of individuals with obesity [5]. In the group of post-MBS individuals, a significant positive correlation was found between anxiety and depression levels and HH.

Lowe and Butryn defend an increasing proportion of human food consumption that appears to be driven by pleasure, not just by the need for calories, as the growing prevalence of global obesity suggests. They described how many individuals experience frequent thoughts, feelings, and urges about food in the absence of any short- or long-term energy deficit. There has been a strong trend in the literature to attribute eating beyond energy needs to a variety of psychological motives (e.g., escape from self-awareness, self-medication, emotional hunger) [6]. Teegarden and Bale suggest that palatable foods may indeed have anxiolytic effects and that ceasing consumption of highly palatable foods could increase stress. These results also potentially provide a whole new way of viewing the nature of “emotional eating” [39].

A study conducted by Yalçin et al. also found a significant positive correlation between HH and anxiety and depression levels [40]. Andreeva et al. described a significant positive correlation of weak intensity between HH and symptoms of anxiety and depression [35]. Fernstrom et al. reported a higher appetite for highly palatable foods in individuals with depression and anxiety [41].

In this investigation, no relationship was observed between adherence to MD and BMI. However, it was found that most individuals did not exhibit good adherence to this dietary pattern. Several factors, including cultural and socioeconomic barriers, can affect patient adherence to MD. The high food cost perception, especially of healthy foods, is one of the reasons for the lack of adherence to MD, alongside the difficulty in storing and preserving perishable foods like fruits and vegetables. Lower incomes, lower education levels, and limited food and health literacy negatively impact patient adherence to MD. Availability and accessibility are also major factors in adherence to diet, both in terms of transportation and the selection of foods in supermarkets [42, 43].

Regarding the relationship between the percentage of weight loss and adherence to MD, no significant association was observed. However, individuals with good adherence to MD showed a higher percentage of weight loss. These results are consistent with those of other studies. Gastaldo et al. found that adherence to MD may be directly related to weight loss, both before and after MBS [11]. Contreras et al. also concluded that higher adherence to MD after surgery was associated with a greater percentage of weight loss [44].

Regarding the correlation between adherence to MD and anxiety and depression levels, no significant relationship was observed. However, in recent years, the role of diet, particularly MD, has been a focus of research and debate in the development of mental disorders [12, 13]. In a clinical trial on MD and mental health, the authors showed a correlation between the reduction of depressive symptoms and adherence to the MD [14].

In this study, only 23.4% of individuals exhibited good adherence to the Mediterranean dietary pattern, which may have affected the relationship between anxiety and depression levels.

In this investigation, no relationship was observed between adherence to MD and HH.

## Strengths and limitations

The strength of this study is that it first evaluated the relationship between adherence to MD and HH.

A limitation of this study is the use of a convenience sample, which limits the extrapolation of the results. Furthermore, a strong limitation of this study beyond the small sample size is that the under-study data were collected by online questionnaires and not by face-to-face interviews. Thus, the collected data may suffer from recall biases.

Recall-bias due to self-reported dietary data is also a limitation. The use of food diaries and biomarkers should be considered in future studies.

The cross-sectional design of the study is another limitation, as it does not allow evaluation of changes within individuals over time. The pre- and post-surgical groups were analyzed as independent cohorts rather than matched pairs, and thus comparisons between these groups should be interpreted cautiously and cannot be considered longitudinal data. Future research should employ longitudinal designs to better assess the evolution of psychosocial factors, hedonic hunger, dietary intake, and weight changes before and after metabolic and bariatric surgery.

Another limitation of this study was the use of two different surgical procedures. Future studies should employ a more homogeneous sample with a single surgical technique.

## Conclusion

In this study, we found higher levels of depression in MBS candidates and a significant association between anxiety, depression, and hedonic hunger (HH). These findings highlight the importance of integrating psychological support into the pre- and post-surgery process. Pre-surgery interventions can help manage anxiety, depression, and HH, potentially improving surgical outcomes, while post-surgery follow-up is essential to address ongoing psychological challenges and support long-term weight management.

Moreover, the low adherence to the Mediterranean Diet observed across the study population underscores the need for tailored nutritional guidance alongside psychological care to optimize patient outcomes.

Future research should explore the complex relationship between psychological factors, HH, food intake, and weight to refine interventions in MBS care, ideally using longitudinal designs to better understand changes over time and inform personalized treatment strategies.

## What is already known on this topic?

Psychosocial factors can influence HH. Moreover, HH can be a predictor of MBS outcomes. MD was used as a model to manage excess weight. However, no evidence exists regarding the relationship between adherence to MD and HH.

## What this study adds?

This study first evaluated the relationship between adherence to MD and HH. In addition, we confirmed a significant positive association between anxiety and depression levels and HH.

## Data Availability

Data are available from the corresponding author.
